# The complete mitochondrial genome of *Chaetodon octofasciatus* (Perciformes: Chaetodontidae) and phylogenetic studies of Percoidea

**DOI:** 10.1080/23802359.2018.1467218

**Published:** 2018-04-27

**Authors:** Kehua Zhu, Li Gong, Zhenming Lü, Liqin Liu, Lihua Jiang, Bingjian Liu

**Affiliations:** a National Engineering Laboratory of Marine Germplasm Resources Exploration and Utilization, Zhejiang Ocean University, Zhoushan, China;; b National Engineering Research Center for Facilitated Marine Aquaculture, Marine science and technology college, Zhejiang Ocean University, Zhoushan, China;; c Laboratory for Marine Fisheries Science and Food Production Processes, Qingdao National Laboratory for Marine Science and Technology, Qingdao, China

**Keywords:** *Chaetodon octofasciatus*, mitogenome, phylogenetic tree

## Abstract

The complete mitochondrial genome of this species was first determined in this study, which is 16,485 bp in length, containing 13 protein-coding genes, 2 rRNA genes, 22 tRNA genes, a putative control region, and 1 origin of replication on the light-strand. The overall base composition includes C(28.2%), A(28.3%), T(27.4%), and G(16.1%). Moreover, the 13 PCGs encode 3796 amino acids in total, 12 of which use the initiation codon ATG except *COI* that uses GTG. Most of them have TAA as the stop codon, whereas *ND3* ends with TAG, and three protein-coding genes (*COII*, *ND4*, and *Cyt*b) ended with the incomplete stop codon represented as a single T. The phylogenetic tree based on the Neighbor Joining method was constructed to provide relationship within Percoidea, which could be a useful basis for management of this species.

The Eightband butterflyfish (*Chaetodon octofasciatus*) is a species of the family Chaetodontidae, which are conspicuously beautiful and abundant animals found on coral reefs worldwide (Hsu et al. 2007). Because of its coral diet, this species often starves when kept in captivity (Pratchett et al. 2008). Considering its ecological importance and commercial value, to gain its molecular information, we described the complete mitogenome of *C. octofasciatus*, and explored the phylogenetic relationship within Percoidea, contributing to phylogenetic studies of Chaetodontidae and provide a new thought for propagation and breeding of this species.

The specimen was collected from Hainan Island, China (18°29′48″N; 109°56′12″E) and stored in laboratory of Zhejiang Ocean University with accession number 20150826BA22. Total genomic DNA was extracted from muscle tissue of individual using the phenol–chloroform method (Barnett and Larson 2012). The calculation of base composition and phylogenetic construction was conducted by MEGA6.0 software (Tamura et al. 2013).

Similar to the typical mitogenome of vertebrates, the mitogenome of *C. octofasciatus* is a closed double-stranded circular molecule of 16,485 bp (GenBank accession No. MH094803), which contains 13 protein-coding genes, 2 ribosomal RNA genes, 22 tRNA genes, and 2 main non-coding regions (Boore 1999; Zhu et al. 2018). The overall base composition is 28.3%, 27.4%, 28.2%, and 16.1% for A, T, C, and G, respectively, with a slight AT bias (55.7%). Most mitochondrial genes are encoded on H-strand except for *ND6* and eight tRNA genes (*Gln*, *Ala*, *Asn*, *Cys*, *Tyr*, *Ser*, *Glu*, *Pro*), which are encoded on the L-strand. 13 PCGs encode 3796 amino acids in total, all of them use the initiation codon ATG except *COI* uses GTG, which is quite common in vertebrate mtDNA (Miya et al. 2001). Most of them have TAA as the stop codon, whereas *ND3* ends with TAG, and three protein-coding genes (*COII*, *ND4*, and *Cytb*) ended with an incomplete stop codon T. The 12S rRNA and 16S rRNA are 950 and 1767 bp, which are both located in the typical positions between tRNA-Phe and tRNA-Leu^(UUA)^, separated by tRNA-Val. The origin of light-strand replication is located in a cluster of five tRNA genes (WANCY) as in other vertebrates (Petrillo et al. 2006), and has the potential to fold into a stable stem-loop secondary structure, with a stem formed by 13 paired nucleotides and a loop of 12 nucleotides. The CR is determined to be 827 bp, by comparing the sequences of the CR with other teleost, three typical domains are observed, including termination-associated sequences, the central conserved sequence block domain and the conserved sequence block domain, which is identical to that in other teleostean mitogenomes (Zhang et al. 2013).

To explore the phylogenetic position of this Eightband butterflyfish, a phylogenetic tree was constructed based on the NJ analysis of 12 PCGs encoded by the heavy strand. The results of the present study supports *C. octofasciatus* has a closest relationship with *C. auripes*, highly supported by a bootstrap value of 100 (Figure 1), which is in accord with the previously reported study (Fessler and Westneat 2007).

**Figure 1.**
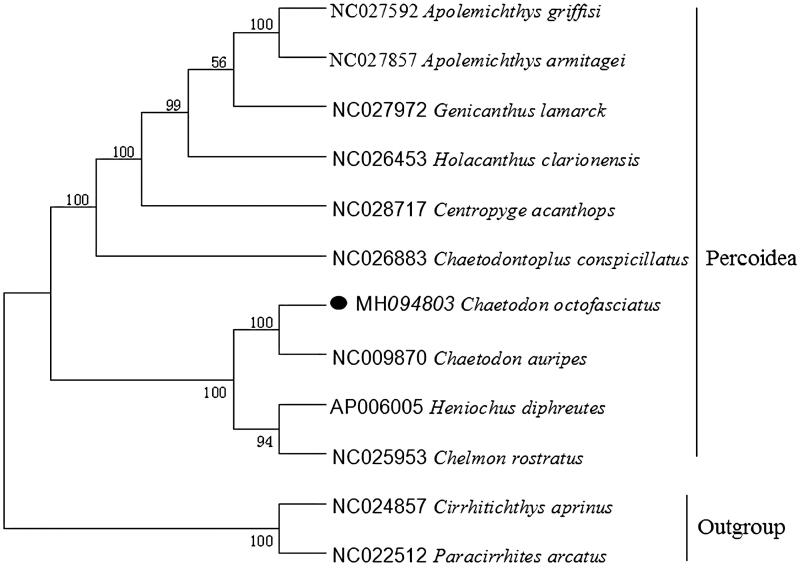
Neighbor Joining (NJ) tree of 10 Percoidea species based on 12 PCGs. The bootstrap values are based on 1000 resamplings. The number at each node is the bootstrap probability. The number before the species name is the GenBank accession number. The genome sequence in this study is labeled with a black spot.

## References

[CIT0001] Barnett R, Larson G. 2012 A phenol-chloroform protocol for extracting DNA from ancient samples. Methods Mol Biol. 840:13–19. 22237516 10.1007/978-1-61779-516-9_2

[CIT0002] Boore JL. 1999 Animal mitochondrial genomes. Nucleic Acids Res. 27:1767–1780. 10101183 10.1093/nar/27.8.1767 PMC148383

[CIT0003] Fessler JL, Westneat MW. 2007 Molecular phylogenetics of the butterflyfishes (Chaetodontidae): taxonomy and biogeography of a global coral reef fish family. Mol Phylogenet Evol. 45:50–68. 17625921 10.1016/j.ympev.2007.05.018

[CIT0004] Hsu KC, Chen JP, Shao KT. 2007 Molecular phylogeny of Chaetodon (Teleostei: Chaetodontidae) in the Indo-West Pacific: evolution in geminate species pairs and species groups. Raffles Bull Zool.14:77–86.

[CIT0005] Miya M, Kawaguchi A, Nishida M. 2001 A case study for moderate-scale evolutionary genomics with 38 newly determined complete mitochondrial DNA sequences. Mol Biol Evol. 18:1993–2009. 11606696 10.1093/oxfordjournals.molbev.a003741

[CIT0006] Petrillo M, Silvestro G, Nocera PPD, Boccia A, Paolella G. 2006 Stem-loop structures in prokaryotic genomes. Bmc Genomics. 7:170 16820051 10.1186/1471-2164-7-170 PMC1590033

[CIT0007] Pratchett MS, Berumen ML, Marnane MJ, Eagle JV, Pratchett DJ. 2008 Habitat associations of juvenile versus adult butterflyfishes. Coral Reefs. 27:541–551.

[CIT0008] Tamura K, Stecher G, Peterson D, Filipski A, Kumar S. 2013 MEGA6: Molecular Evolutionary Genetics Analysis version 6.0. Mol Biol Evol. 30:2725–2729. 24132122 10.1093/molbev/mst197 PMC3840312

[CIT0009] Zhang H, Zhang Y, Zhang X, Song N, Gao T. 2013 Special structure of mitochondrial DNA control region and phylogenetic relationship among individuals of the black rockfish, Sebastes schlegelii. Mitochondrial DNA. 24:151–157. 23072475 10.3109/19401736.2012.731401

[CIT0010] Zhu K, Lü Z, Liu L, Gong L, Liu B. 2018 The complete mitochondrial genome of *Trachidermus fasciatus* (Scorpaeniformes: Cottidae) and phylogenetic studies of Cottidae. Mitochondrial DNA Part B. 3:301–302. 10.1080/23802359.2018.1445480 PMC7799978 33474152

